# Achilles Tendon Repair by Decellularized and Engineered Xenografts in a Rabbit Model

**DOI:** 10.1155/2019/5267479

**Published:** 2019-08-29

**Authors:** Marta Bottagisio, Daniele D'Arrigo, Giuseppe Talò, Matilde Bongio, Marco Ferroni, Federica Boschetti, Matteo Moretti, Arianna B. Lovati

**Affiliations:** ^1^Laboratory of Clinical Chemistry and Microbiology, IRCCS Istituto Ortopedico Galeazzi, Milan 20161, Italy; ^2^Cell and Tissue Engineering Laboratory, IRCCS Istituto Ortopedico Galeazzi, Milan 20161, Italy; ^3^Department of Chemistry, Materials and Chemical Engineering Giulio Natta, Politecnico di Milano, Milan 20133, Italy; ^4^Regenerative Medicine Technologies Lab, Ente Ospedaliero Cantonale, Lugano 6900, Switzerland; ^5^Cardiocentro Ticino, Lugano 6900, Switzerland

## Abstract

Tendon tissue ruptures often require the replacement of damaged tissues. The use of auto- or allografts is notoriously limited due to the scarce supply and the high risks of immune adverse reactions. To overcome these limitations, tissue engineering (TE) has been considered a promising approach. Among several biomaterials, decellularized xenografts are available in large quantity and could represent a possible solution for tendon reconstruction. The present study is aimed at evaluating TE xenografts in Achilles tendon defects. Specifically, the ability to enhance the biomechanical functionality, while improving the graft interaction with the host, was tested. The combination of decellularized equine-derived tendon xenografts with or without the matrix repopulation with autologous bone marrow mesenchymal stem cells (BMSCs) under stretch-perfusion dynamic conditions might improve the side-to-side tendon reconstruction. Thirty-six New Zealand rabbits were used to create 2 cm long segmental defects of the Achilles tendon. Then, animals were implanted with autograft (AG) as the gold standard control, decellularized graft (DG), or in vitro tissue-engineered graft (TEG) and evaluated postoperatively at 12 weeks. After sacrifice, histological, immunohistochemical, biochemical, and biomechanical analyses were performed along with the matrix metalloproteinases. The results demonstrated the beneficial role of undifferentiated BMSCs loaded within decellularized xenografts undergoing a stretch-perfusion culture as an immunomodulatory weapon reducing the inflammatory process. Interestingly, AG and TEG groups exhibited similar results, behaved similarly, and showed a significant superior tissue healing compared to DG in terms of newly formed collagen fibres and biomechanical parameters. Whereas, DG demonstrated a massive inflammatory and giant cell response associated with graft destruction and necrosis, absence of type I and III collagen, and a higher amount of proteoglycans and MMP-2, thus unfavourably affecting the biomechanical response. In conclusion, this in vivo study suggests a potential use of the proposed tissue-engineered constructs for tendon reconstruction.

## 1. Introduction

The increasing number of traumatic events resulting in tendon loss and/or degenerative tendinopathy causing complicated ruptures often requires the replacement of the damaged tissue, especially in elderly patients and athletes. As a matter of fact, the tendon self-repairing ability is poor due to both the scarce presence and the metabolic activity of resident cells that cannot properly guide the healing process required to restore the native tissue function [1, 2]. The surgical procedures commonly performed to treat tendon ruptures with auto- or allografts might not guarantee the successful restoration of the tendon function and, besides that, relapses are frequent [3]. Furthermore, the use of either auto- or allografts is notoriously limited due to the scarce supply and the high risks connected to immune adverse reactions or disease transmission following the tendon substitution, respectively. In this context, the reconstruction of the tissue with synthetic grafts has been extensively investigated [4], together with several tissue engineering strategies to enhance the regenerative potential of implanted grafts [5]. However, the use of synthetic biomaterials has been proposed with questionable results in terms of biocompatibility and biomechanical properties [6].

Undeniably, there is no better way to substitute a damaged tissue with a homologous healthy structure. Hence, the ideal graft for tendon substitution is a nonimmunogenic, easily available extracellular matrix (ECM) with mechanical properties resembling those of the native tissue offering a valid scaffold to host cell ingrowth [6]. In this regard, the decellularization of xenografts appeared to be a promising strategy for the treatment of tendon tears and ruptures. In particular, equine tendon biomechanical properties, large quantity supply, and less uncontrolled cross-species diseases compared to other animals (swine, bovine, etc.) offer several advantages over both human-derived and synthetic scaffolds [7]. Previous studies demonstrated that the decellularization of equine tendons appeared to be a promising strategy for the generation of an acellular matrix lacking in antigens by preserving matrix and offering cells a natural environment [7, 8]. However, an implanted decellularized matrix will take a long time to eventually be repopulated with host resident cells and to become a vital biological scaffold. Hence, a stem cell-based approach might be exploited to encourage the decellularized matrix colonization while guiding the tendon regeneration [9]. Several researches demonstrated the ability of both human and animal bone marrow-derived mesenchymal stem cells (BMSCs) to differentiate into tenocyte-like cells in response to chemical and physical stimuli in vitro as well as in vivo [10–12]. To support the cell alignment, differentiation, and ECM deposition within the acellular matrix, an oscillating stretch-perfusion bioreactor (OSPB) was recently designed and validated in vitro for the functionalization of cell-seeded decellularized equine tendons [13]. In this study, it has been described how the use of OSPB confers to undifferentiated cells the mechanical stimuli required to highly orient both seeded cells and the newly deposed collagen while preserving the biomechanical properties of the scaffolds, thus guaranteeing a high cell engraftment within the engineered constructs [13]. Despite the promising results obtained in vitro, the use of an animal model to test the feasibility of the proposed regenerative therapy for injured tendons is mandatory. In order to identify the most appropriate animal model to verify the proposed scientific hypothesis, a review of the literature evaluating animal models and treatments for the reconstruction of Achilles and flexor tendons has been recently carried out [14]. From this analysis, rabbit resulted the most frequently used animal species due to the intrinsic advantages, such as the easy handling and appropriate tendon size and biomechanical properties resembling those of human flexor tendons [14, 15]. Considering the excellent above-mentioned results obtained in vitro, the present study is aimed at testing novel biofabricated tendon xenografts in a rabbit model of the Achilles tendon full transection. Specifically, the ability to increase the biomechanical functionality, while improving the tendon graft interaction with the host, will be tested and compared to standard autografts as the control. The combination of a complete decellularized equine xenograft together with autologous cell repopulation of the matrix under dynamic conditions might be a promising approach to repair tendon ruptures in clinics.

## 2. Materials and Methods

### 2.1. Ethics Statement

The whole study was approved by the Institutional Animal Care and Use Committees (IACUC) of the University of Modena and Reggio Emilia (Permit N. 915/2016-PR). The animals were housed in the Institute's Animal Care Facilities that meet international standards. The University of Modena and Reggio Emilia adheres to the principles set out in the following laws, regulations, and policies governing the care and use of laboratory animals: Italian Governing Law (D. legs 26/2014) and EU directives and guidelines (EEC Council Directive 2010/63/UE). The animals were regularly checked by a certified veterinarian responsible for health monitoring, animal welfare supervision, experimental protocols, and procedure revision. All surgeries were performed under general anaesthesia, and all efforts were made to minimize animal suffering.

### 2.2. Study Design

Thirty-six male New Zealand white rabbits (Oryctolagus cuniculus; body weight 3.68 ± 0.55 kg, 15 weeks old) were used in this study (Envigo RMS Srl, S. Pietro al Natisone, Udine, Italy). All animals were singly caged and maintained under controlled conditions: room temperature 20-22°C, 45% humidity, 15 air changes per hour, and a light/dark cycle of 12 h. Rabbits had free access to water and standard food pellets ab libitum (diet 2RB15, Mucedola s.r.l., Settimo Milanese, Italy). Animals were acclimatized for two weeks before surgery; thereafter, the subjects were randomly divided into three groups: (1) autograft (*n* = 12) (AG, reversed autograft), (2) decellularized graft (*n* = 12) (DG), and (3) in vitro tissue-engineered graft (*n* = 12) (TEG). Each animal underwent surgical treatment of one hind limb, and the contralateral limb served as the native tendon (NT).

### 2.3. Anaesthesia and Postoperative Treatments

All the procedures on rabbits were performed under general anaesthesia. Rabbits were anaesthetized with an intramuscular cocktail of ketamine hydrochloride (30 mg/kg; Imalgene 1000, Merial, Milan, Italy) and xylazine (4 mg/kg; Sedaxylan 2%, Dechra Veterinary Products Srl, Barcelona, Spain) and maintained with isoflurane (1-3%; Isoflurane Vet, Merial, Milan, Italy) in oxygen by a face mask. The marginal ear vein was catheterized, and animals were hydrated with sterile solution. In the postoperative pain control, hydrochloride buprenorphine (0.03 mg/kg; Buprenodale, Dechra Srl, Turin, Italy) was intravenously injected. Other postoperative treatments included subcutaneous administrations of flunixin meglumine (1 mg/kg; Finadyne, MSD Animal Health Srl, Segrate, Italy) and parenteral administrations of marbofloxacin (2 mg/kg, Marbocyl, Vetoquinol Italia Srl, Bertinoro, Italy) for 5 days. After 12 weeks of follow-up, animals were anesthetized as aforementioned, then euthanized with an overdose of pentobarbital-sodium (90 mg/kg, Pentothal Sodium, Intervet Productions Srl, Aprilia, Italy) in order to proceed with the ex vivo analyses.

### 2.4. Bone Marrow Harvest and rbBMSC Culture

Rabbits included in the TEG group underwent a bone marrow harvest to retrieve autologous rbBMSCs. Briefly, under general anaesthesia, animals were placed in ventral recumbency with the hind limbs flexed underneath. The dorsal area was shaved and disinfected for the procedure. The iliac crest was identified, a small skin incision was performed, and a bone marrow aspiration needle (Jamshidi biopsy needle 7 cm; 18 G; Byopsibell, Modena, Italy) was introduced through the marrow cavity by clockwise and counterclockwise rotations. Then, the needle stylus was removed and a 10 ml syringe containing 5000 UI/ml sodium heparin (Pharepa, PharmaTex Italia, Milan, Italy) was connected to the luer-lock. After the heparin injection, about 5 ml of bone marrow was immediately aspirated and mixed gently, the syringe was capped, and the samples were stored at 4°C to be processed within few hours.

The skin incision was closed with monofilament nylon sutures (Monosof 3-0, Medtronic, Milan, Italy) and rabbits were recovered from anaesthesia. Following, rbBMSCs were isolated by centrifuging the bone marrow samples at 400 xg for 5 min, removing the supernatant, collecting the buffy layer of mononuclear cells, and washing twice in phosphate-buffered saline (PBS, Gibco). Subsequently, the obtained pellet was suspended in a complete medium (CM) composed of a high glucose Dulbecco's modified Eagle's medium containing 4.5 g/l glucose (DMEM-HG) (Gibco), 10% foetal bovine serum (FBS, HyClone), 100 U/ml penicillin-streptomycin, 2 mM L-glutamine, 1% sodium pyruvate, 1% HEPES (all from Gibco), and 10 ng/ml of basic fibroblast growth factor (bFGF, Invitrogen, Milan, Italy) and plated in cell culture flasks. Rabbit BMSCs were selected by plastic adherence [16] and further expanded by seeding cells at a density of 6000 cells/cm^2^ at 37°C, 5% CO_2_) in CM. Fresh medium was changed twice a week, and cells were subcultured after reaching 90% of confluence after about 12 days, as described elsewhere [12]. Second-passage cells were frozen until use.

### 2.5. Cell Reseeding of Decellularized Xenografts and Dynamic Culture in a Bioreactor

Xenografts obtained by the decellularization of equine superficial digital flexor tendons were measured for the DNA content (17.95 ng/mg ± 7.87 SD dry weight) to assess the efficiency of cell removal, and then, they were terminally sterilized as widely described in our previous studies [8, 17]. Autologous rbBMSCs were thawed at the second passage, expanded, and seeded onto and into the decellularized xenografts at the third passage. The decellularized xenografts were sized at 0.5 cm width × 4 cm length × 0.3 cm thickness, and their surfaces seeded with 50,000 cells/cm^2^ resuspended in 30 *μ*l of CM and incubated for 2 h to allow the cell attachment. Moreover, twelve injections of 20,000 rbBMSCs/20 *μ*l were performed perpendicular to the decellularized matrix fibres throughout their length. Afterwards, the CM was added to completely cover the constructs, and rbBMSCs were cultured on the surface of decellularized xenografts in static conditions for 72 h. Then, the cell-seeded decellularized xenografts were mounted in the culture chamber of a custom-made bioreactor and dynamically cultured for 7 days based on our previous study [13]. Finally, the tissue-engineered xenografts were implanted as tendon substitutes, after verifying the viability of reseeded cells through the Live&Dead assay (data not shown).

### 2.6. Surgical Procedure to Implant Tendon Grafts

**Figure 1:**
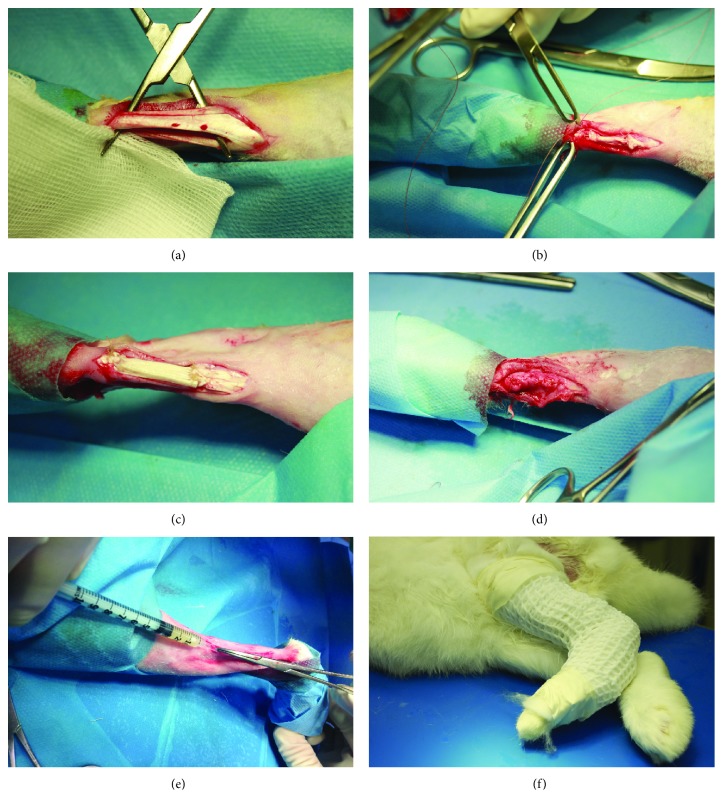
Surgical approach. (a) Achilles tendon complex exposure (NT). (b) Tenotomy bridged with an autograft (AG). (c) Implantation of the decellularized graft (DG). (d) A representative picture of the tendon sheath closure. (e) Injection of autologous rbBMSCs within the tissue-engineered graft (TEG). (f) Application of the plaster cast at 150° flexion.

Under general anaesthesia and in ventral recumbency, one hind limb of each subject was shaved and disinfected as standard. The Achilles tendon complex was exposed by a midline skin, subcutis, and the fascia incision at 0.5 cm distal to the gastrocnemius muscle and 0.5 cm above the calcaneus (Figure 1(a)). A 2 cm long segmental resection was created in the midsubstance of the Achilles tendon, including the medial and lateral gastrocnemius. The segmental resected tendons were used as reversed autografts in the AG group. Then, immediately after tenotomy, and according to the experimental group, the tendon defect was bridged end-to-end with AG (Figure 1(b)), DG, or TEG constructs—shaped to fit in the defects—with a 4-0 absorbable material (Polysorb, Medtronic) using the modified Kessler technique that connected the stumps of the native tendon and the specific implanted graft (Figure 1(c)). The edges of the tendon were also sutured by running epitendinous stitches with the same suture material, based on the technique described elsewhere [18, 19]. The plantaris tendon, also known as flexor digitorum superficialis (FD), was left intact as an internal splint. The paratenon was closed with a 5-0 absorbable material (Polysorb, Medtronic) (Figure 1(d)), and the skin sutured as standard with a nonabsorbable suture (Monosof 3-0, Medtronic, Milan, Italy). After suturing the paratenon, the TEG group also received a local injection of autologous rbBMSCs (1 × 10^6^ cells/kg b.w.) with the purpose to ameliorate the tissue healing and to modulate the host response to the xenograft after implantation (Figure 1(e)). After surgery, an antimicrobial ointment (Hyalosilver, Fidia Pharmaceutics, Abano Terme (PD), Italy) was sprayed on the wound and the treated leg was wrapped up with a soft padded bandage before putting in a plaster cast (X-Lite, A3-MED, Zola Predosa, Italy). The cast extending up to the ankle joint was applied for 10 days at 150° flexion to prevent overloading (Figure 1(f)) [20]. Animals were free to move within the cage without restrictions. After 10 days, the plaster cast was removed, and under general anaesthesia, high frequency ultrasounds were performed in longitudinal planes with a linear probe of 18 MHz (MyLab™One, Esaote S.p.A., Genoa, Italy) to monitor the status of the tendon replacement in all animals. The contralateral healthy tendon was evaluated as controls (NT). In particular, the attachments of the grafts to the proximal and distal ends of the tendon were assessed. Then, a soft cast (Vetrap, 3M, Pioltello, Italy) was worn for 2 weeks and then definitively removed. After 12 weeks, animals were sacrificed and tendon specimens were harvested from the calcaneus to the muscle aponeurosis junction. In particular, for each experimental group, six explants were processed for histological analyses (except for the AG group *n* = 5) and six explants were dry-stored at -80°C for both biochemical and biomechanical tests.

### 2.7. Biochemical Analysis for Glycosaminoglycan Quantification

Small segments of tendon explants were weighted, minced, and digested in 2 mg/ml proteinase K (pH 7.6) (Sigma-Aldrich, Milan, Italy) at 56°C for 16 h under agitation. Digested samples were centrifuged at 10000 xg for 10 min at RT, and the supernatant was collected for analyses. The quantification of glycosaminoglycans (sGAG) was performed using the 1,9-dimethylmethylene blue (DMMB) dye-binding assay (Sigma-Aldrich, Milan, Italy). Briefly, samples were incubated in 40 mM glycin/NaCl (pH 3.0) with 16 mg/ml DMMB at RT. The sGAG concentration was determined by reading the absorbance at 500 nm (Perkin Elmer Victor X3 microplate reader) and comparing the results to a standard curve of chondroitin sulphate (Blyscan, Biocolor, Magenta, Italy). Data from the sGAG content analysis were normalized on the sample dry weights.

### 2.8. Histological Analysis

Being explanted tendons very long, before fixation and inclusion, each sample was divided for the entire width at its middle thirds into a proximal and a distal portion, representing the areas of transition between the native tendons and the grafts. Then, the specimens were fixed in 10% neutral buffered formalin for 24 h and dehydrated in alcohol scale before embedding in paraffin and cutting into 4 *μ*m longitudinal sections. The slides were stained with Alcian Blue (AB). Photomicrographs of the tissue were captured through an Olympus IX71 light microscope and an Olympus XC10 camera (Japan). Three independent, blinded examiners assessed the tendon histopathology according to a modified semiquantitative grading score, as proposed by others [20, 21]. In particular, the following parameters were evaluated: organization of the extracellular matrix (0-2), cellularity (0-2), cell alignment (0-2) and distribution (0-1), the morphology of the cell nuclei (0-2), organization of repair tissue of the tendon callus (0-2), transition from defect to normal tissue (0-2), vascularization (0-1), degenerative changes (0-3), inflammation (score 0-1), and proteoglycan content (0-1). The maximum score (19) represented the best outcome in terms of tendon tissue integrity, and the minimum value (0) represented the worst condition.

### 2.9. Immunohistochemistry for Type I and III Collagen Detection

To evaluate the distribution of type I and III collagen, immunohistochemistry was performed on deparaffinised sections. The sections were incubated with monoclonal primary antibodies for anticollagen type I (1 : 200 dilution) and type III (1 : 2000 dilution) (Sigma-Aldrich Corporation, Saint Louis, MO) for 1 h at RT. Then, sections were exposed to a biotinylated anti-mouse secondary antibody (1 : 200 dilution; Vinci Biochem, Vinci, Florence, Italy) for 30 minutes. The signal was detected by means of the streptavidin-biotin method coupled with the 3′-Diaminobenzidine (DAB) chromogen system (Vinci Biochem). Sections were counterstained with haematoxylin, microscopically analysed. Negative control was carried out by omitting the primary antibodies. Positive control staining was obtained from rabbit ear tissues.

### 2.10. Histomorphometric Analysis by Means of Stereological Method

The presence of sulphate proteoglycans was assessed in AB-stained sections by means of histomorphometry. In particular, the histological quantification was performed using the ImageJ 1.45 software (open source: http://rsbweb.nih.gov/ij), as described elsewhere [22]. Specifically, the area occupied by proteoglycans within the region of interest (ROI) was analysed after removing both the white and the collagen matrix areas by means of a colour threshold. Data are reported as the percentage of the total ROI of the tissue.

### 2.11. Gelatin Zymography

The proteolytic activity of matrix metalloproteinase 2 (MMP-2) and 9 (MMP-9) was evaluated through gelatine zymography. Proteins were extracted from tendon specimens as previously described [23, 24]. Briefly, 50 mg of tendon was minced and incubated with 500 ml of extraction buffer composed of 10 mM cacodylic acid (pH 5.0), 0.15 M NaCl, 1 *μ*M ZnCl_2_, 20 mM CaCl_2_, 1.5 mM NaN_3_, and 0.01% Triton X-100 (all from Sigma-Aldrich) at 4°C for 24 h. Then, the extraction buffer was collected and refreshed, and samples were treated for further 24 h. Before protein separation, the pH of the collected extraction buffer was raised to 7.5 by adding a 1 M solution of Tris (pH 8.0, Sigma-Aldrich) and the extracted proteins were quantified using the Pierce™ BCA Protein Assay Kit (Thermo Fisher Scientific). After that, 0.12% gelatine precasted gels (Novex™, Life Technologies) were loaded with 20 *μ*g of proteins and run at 125 V until the dye front ran off the gel. Gels were then removed from the cassettes and incubated first in renaturing buffer (Novex™, Life Technologies) for 30 min with gentle agitation and then similarly in developing buffer for 30 min. Subsequently, the developing buffer was decanted and refreshed, and gels were incubated overnight at 37°C for maximum sensitivity. Prior to staining, gels were rinsed three times with dH_2_O to remove any remaining of sodium dodecyl sulphate (SDS) and immersed in Coomassie blue staining solution (SimplyBlue™ SafeStain, Life Technologies) for 15 min. Metalloproteinases activity was visualized as clear bands in the blue gel. Images of the gels were acquired by means of the ChemiDoc™ Imaging System (Bio-Rad), and band intensity was measured using ImageJ software (https://imagej.nih.gov/ij/). Data are reported as peak area values normalized on positive controls (FBS, HyClone).

### 2.12. Biomechanical Analysis

At 12 weeks postsurgery, the specimens were harvested proximally from the muscle tendinous aponeurosis to distally to the calcaneus bone insertion and then stored at -80°C until processing. Biomechanical tests were performed at room temperature on the explants using an electromechanic testing machine (MTS Synergie, Eden Prairie, MN, USA), equipped with a load cell of 1 kN. Briefly, after thawing, to prevent the slippage of the samples, the calcaneal insertion and muscle-tendon transition were wrapped in sheets of sandpaper, glued with cyanoacrylate, and clamped between the grips. During the biomechanical test, the samples were continuously irrigated with PBS to prevent dehydration. Six preconditioning cycles till 10% of initial length were applied to every sample before the tests. Then, specimens were ultimately loaded to failure at a uniaxial tension at 1%/s strain rate following the protocols of our previous studies [8, 17]. The elastic modulus at high strains (EM, MPa), evaluated as the slope of the final linear portion of the stress-strain curve, and the failure stress (MPa) of the tendon hyperelastic response were derived from the failure tests. The dataset was analysed by normalizing the obtained results on the cross-sectional area of each specimen.

### 2.13. Statistical Analysis

Statistical analyses were performed with GraphPad Prism 5 software (GraphPad Software Inc., La Jolla, USA). The Shapiro-Wilk test was used to assess the normal distribution of data.

Data obtained for normal distributed values were analysed with one-way analysis of variance (ANOVA) and coupled with Bonferroni's post hoc test; for nonparametric data, Kruskal-Wallis test coupled with Dunns' post hoc was performed. Values of *p* < 0.05 were considered statistically significant. Data are expressed as mean ± standard error (SE). For the in vivo study, the sample size was calculated by power analysis assuming the 80% power to detect differences in the presence or in the absence of transplanted xenograft integration within the native tissue, using a two-sided *t*-test with an *α* error = 0.05 (G^∗^Power 3.1 software, Düsseldorf, Germany). The interrater reliability of the examiners' scores for histopathological evaluations was calculated with intraclass correlation coefficient (ICC): ICC = 1, perfect reliability; ICC > 0.75, excellent reliability [25].

## 3. Results

### 3.1. Clinical Examination, Ultrasound Imaging, and Gross Appearance

Three weeks after surgery, one rabbit of the AG group was suppressed following a trauma occurred in the cage that compromised the animal mobility and determined the development of severe bedsores. Therefore, this subject was excluded from the study. None of the other animals included in any group died or had clinical evidence of local or systemic infection, such as local inflammation (hyperaemia or exudation), diarrhoea, and behavioural alterations.

All wounds healed well and completely 10 days after surgery. At that time, all animals were able to ambulate with a full range of motion after the cast removal.

**Figure 2:**
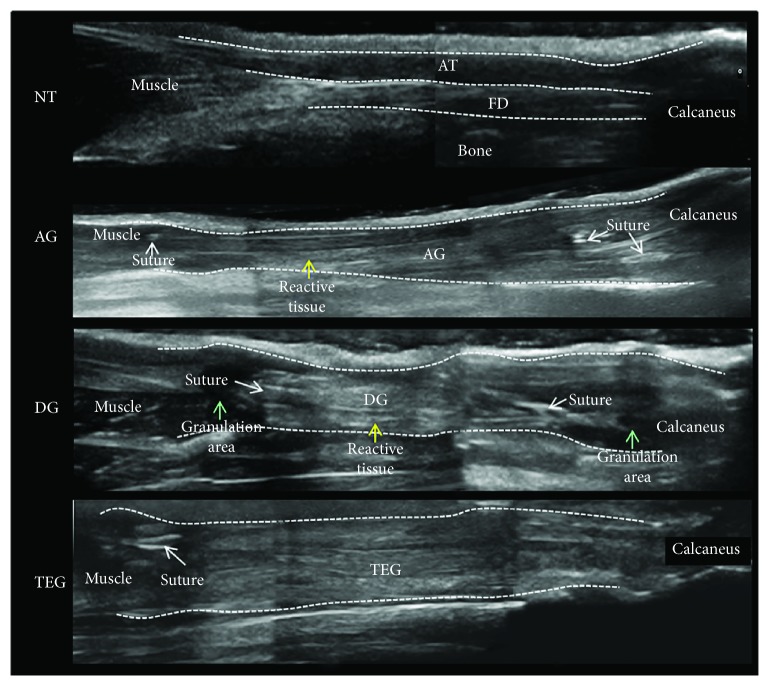
High-frequency ultrasound images of the longitudinal plane of rabbit Achilles tendon complex (AT) and flexor digitorum superficialis (FD) at proximal, mid-, and distal section of the native tendon (NT) and treated groups (AG, DG, and TEG).

At the ultrasound examination along the longitudinal plane (Figure 2), all treated groups showed an enhanced echogenicity in their midthird section. The DG group showed a greater hyperechogenicity compared to the AG and TEG. Moreover, in the DG group, the elongated, linear shape of the tendon was lost and a great presence of reactive tissue together with hypoechogenic granulation tissue was found in the transition zones. Again, in the DG group, the heterogeneity of greyscale ultrasound revealed the presence of swollen tissue within and around the implanted graft, as well as unordered fibre structure. Finally, the presence of adhesions between the implanted graft and the superficial plane of the skin was evident in the DG group. Despite both the detection of suture material and hypoechogenic areas next to the repair site were recognizable, the implanted zones were homogeneous in their slightly enhanced echogenicity in AG and TEG. More importantly, the ultrasound analysis identified an enlargement of the treated area in all groups that was more detectable in the TEG group compared to both the DG and the AG.

At 12 weeks postrepair, the treated tendons appeared longer compared to the NT on gross examination (data not shown). DG and TEG groups exhibited relatively enlargement in the middle third of the flexor tendon complex compared to both AG and NT. In particular, the measurement of the explanted specimens demonstrated greater enlargement of the TEG compared to both the DG and NT groups, as shown in Table 1, thus confirming data recorded by the ultrasonography.

### 3.2. Glycosaminoglycan Content

**Figure 3:**
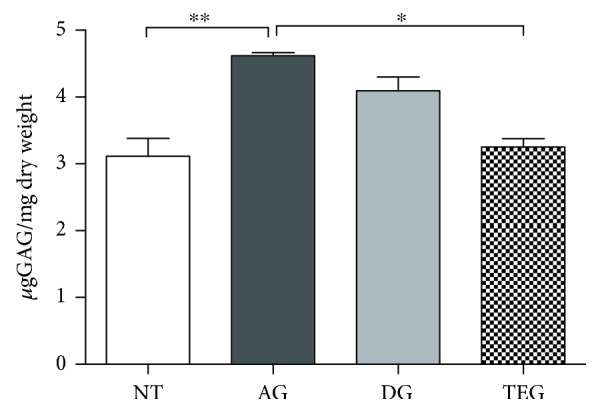
Glycosaminoglycan content of the experimental groups (*μ*g GAGs/mg dry weight). Data are reported as mean ± SE. Nonparametric one-way ANOVA with Dunn's correction showed a significant difference between AG and TEG or NT *p* < 0.05 and *p* < 0.01, respectively. No differences were found between DG or TEG and NT.

A greater amount of sulphated glycosaminoglycans was found in all the treated groups. In particular, the AG group had higher sGAG content compared to the NT and TEG groups for *p* < 0.01 and 0.05, respectively. No significant differences were found between DG or TEG and the NT (Figure 3).

### 3.3. Qualitative and Semiquantitative Histopathological Evaluations and Immunohistochemical Analysis

**Figure 4:**
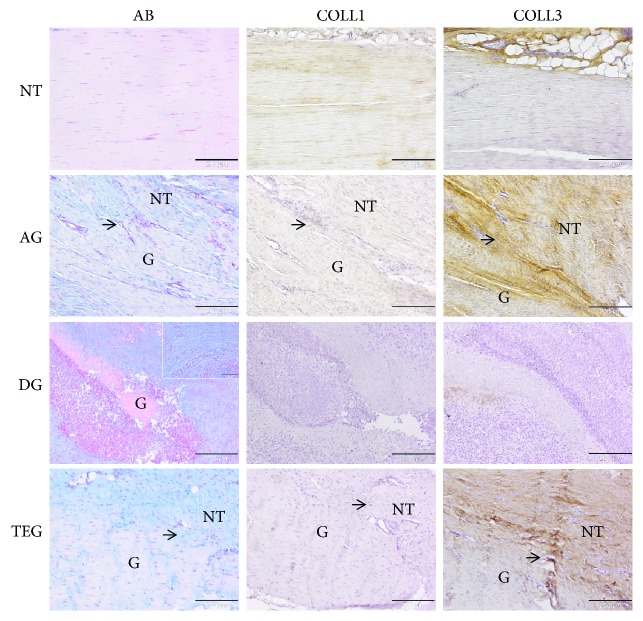
Representative histological and immunohistochemical microphotographs of the native tendon (NT) and treated groups (AG, DG, and TEG) at 12 weeks. The panel reports the Alcian Blue staining (AB) and immunohistochemistry for type I and III collagen (COLL1, COLL3). The black arrows show the transition zones between the native tendon (NT) and the grafts (G). In the DG group, the granulation tissue and inflammatory tissue impede the identification of the transition zone; G letter identifies a portion of the transplanted graft. Magnification 100x, scale bar 200 *μ*m. Detailed box, magnification 200x, scale bar 100 *μ*m.

The qualitative histology and immunohistochemistry for type I and III collagen are depicted in Figure 4.

In all the samples of the NT group, parallel wavy bundles of dense type I collagen fibres and absence of type III collagen were detected. Uniaxial and homogenous distribution of elongated tenocytes was found as well as no abnormalities in terms of degenerative tissue, vascularization, or inflammatory reaction were present.

In the AG group, the recognizable transition zone presented a heterogeneous appearance of compact and mildly disorganized collagen fibres mainly consisting in type III collagen. The AB staining highlighted the presence of slightly enhanced proteoglycans and a moderate increase cell density, where rounded cells, irregularly arranged, were interposed between elongated cells. The neoangiogenesis was a little increased and associated with few lymphocytes along with the presence of a moderate formation of oedema.

**Figure 5:**
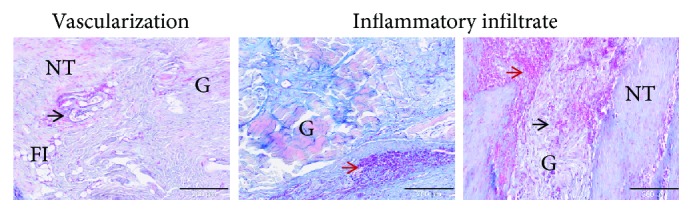
Representative histological panel of the increased vascularization and inflammatory infiltrated found in the DG group at 12 weeks. The panel reports the Alcian Blue staining (AB). The black arrows show the enlargement and increasing small and large capillary vessels in the transition zone and within the implanted graft (G). The red arrows show the huge inflammatory infiltration both near the vessels and in the transition zone between the native tissue (NT) and the graft (G). The fatty infiltrate (FI) is also detectable. Magnification 100x, scale bar 200 *μ*m.

In the DG group, the evident transition zone presented granulation tissue intermingled with proteoglycans and newly formed fibrocartilage tissue (Figure 4, small box). A huge amount of inflammatory cells was present (Figure 5); in particular, foreign body giant cells were detectable along with a significantly increased cell number, which showed a disorganized pattern. Areas of graft destruction were present with noticeable necrotic changes, correlating with the ultrasound examination. The entire tissue structure was lost and the matrix depicted a barely present type I collagen and a poor amount of type III collagen deposed in small areas within the inflammatory reaction (Figure 4). Moreover, an increased number of small and large capillaries was detected in this group (Figure 5).

In the TEG group, despite the presence of a mild proteoglycan deposition and some degenerative changes, such as fat infiltrating tissue and a hypervascularization, the transition zone presented a variation between crimped and disorganized collagen fibres. Some sparse inflammatory cells were present, thus potentially altering the deposition of type I collagen. However, type III collagen was identifiable as a response to resident cell deposition of newly formed matrix. Indeed, after 12 weeks of healing process (second proliferation stage), tendon fibroblasts from the epitenon and the synovial sheath and intrinsic tenocytes from the endotenon are recruited to the injury site to produce collagen type III and ECM components (e.g., proteoglycans) to create an initially unorganized ECM. Then, the production of collagen type III begins to be further replaced by stronger collagen type I over time.

The interrater reliability of the histological score performed by three blinded observers was excellent with an ICC 0.93 (95% CI “0.8865 to 0.9599”).  Table 2 reported the mean values of each single parameter of the modified Stoll's score evaluated by the three blinded observers for the treated groups AG (*n* = 5), DG (*n* = 6), and TEG (*n* = 6).

**Figure 6:**
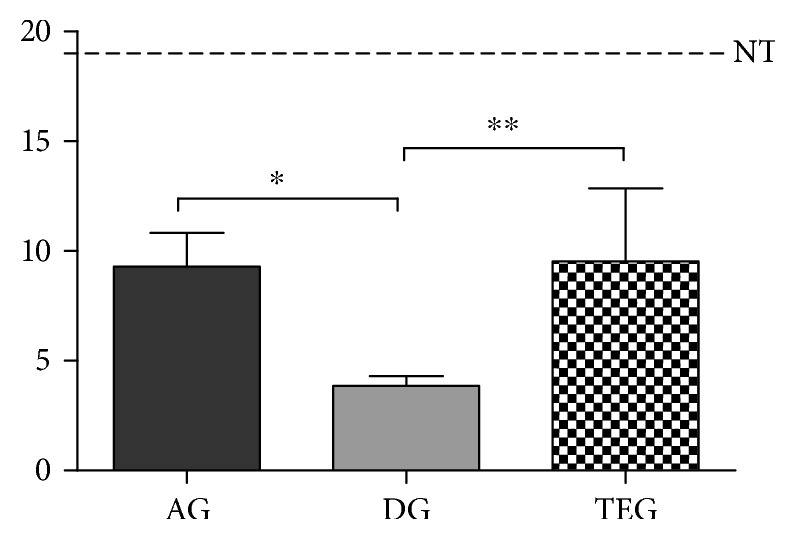
Histopathological score of the treated groups at 12 weeks. No differences were found between AG and TEG, while a significant increase was measured in AG and TEG compared to AG for *p* < 0.05 and *p* < 0.01, respectively.

The overall sum of the histopathological score showed a significant difference between DG and AG or TEG for *p* < 0.05 and *p* < 0.01, respectively. No differences were found between AG and TEG. As expected, all the treated groups had a score significantly different compared to the native tendon (*p* < 0.001) (Figure 6).

### 3.4. Quantitative Assessment of Sulphate Proteoglycans

**Figure 7:**
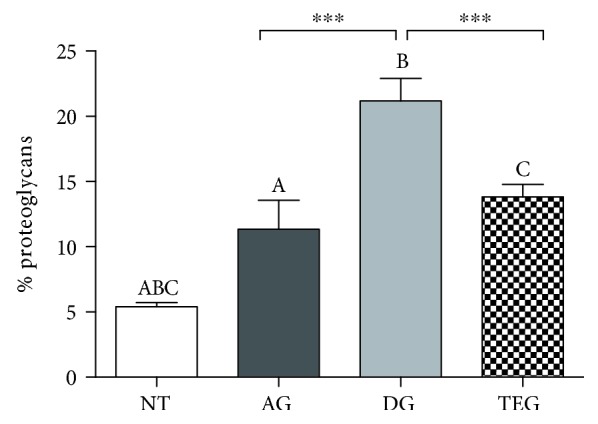
Histomorphometric analysis of proteoglycan content at 12 weeks. No differences were found between AG and TEG, while a significant increase was measured in DG compared to both AG and TEG for *p* < 0.001.

The histomorphometric analysis of the proteoglycan content showed an increased amount in all the treated groups compared to the native tendons. Particularly, the presence of proteoglycans in the AG group was different from NT for *p* < 0.05, and both DG and TEG depicted a higher increase compared to NT (*p* < 0.001). More importantly, no significant differences were found for the proteoglycan content between AG and TEG, while a significant increase of these mucoproteins was present in the DG group compared to both AG and TEG (*p* < 0.001) (Figure 7).

### 3.5. Matrix Metalloproteinase Detection

**Figure 8:**
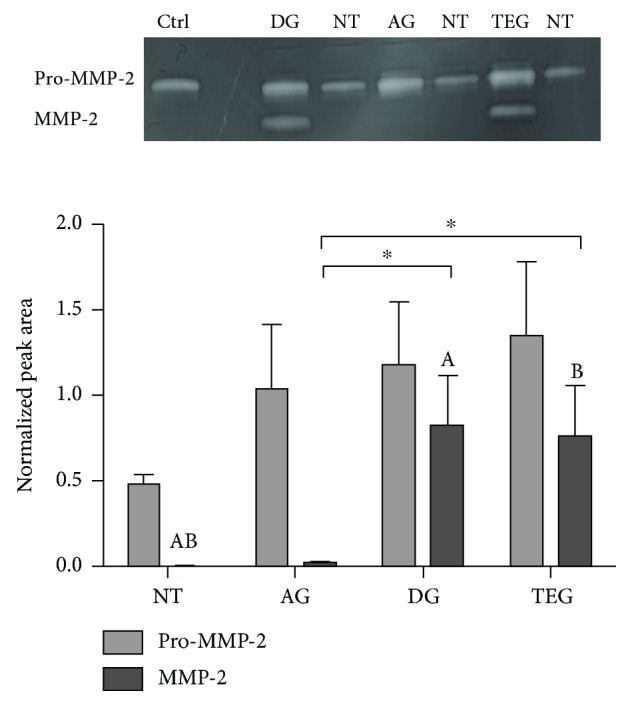
Expression and enzymatic activity of MMP-2. Bands at 72 and 62 kD represent the latent (Pro) and the active form of MMP-2, respectively. The values of the normalized peak areas on the reference control (Ctrl) are reported as the mean ± SEM (*n* = 6). There was no significant change in 72 kD bands among the treated tendons and between treated tendons and NT. In 62 kD bands, the activity was significantly higher in DG and TEG compared to NT for *p* < 0.01 (A) and *p* < 0.001 (B). A significantly higher activity was detected between DG and TEG compared to AG (*p* < 0.05).

The zymography method allowed the identification of protease enzymes with gelatinolytic activity depicted in the bands reported in Figure 8. The 72 kD band represents the latent form of MMP-2 (Pro-MMP-2), while the migrating 62 kD band represents the activated form of the MMP-2 enzyme. There were no significant changes in the intensity of Pro-MMP-2 bands among the treated tendons and between treated tendons and NT. Differently, the activated form of MMP-2 showed a significantly higher activity in DG and TEG compared to NT for *p* < 0.01 (a) and *p* < 0.001 (b). A significantly higher activity was also detected between DG and TEG compared to AG (*p* < 0.05). No results were obtained for MMP-9 due to the poor quality of barely visible bands in the gels.

### 3.6. Biomechanical Testing

**Figure 9:**
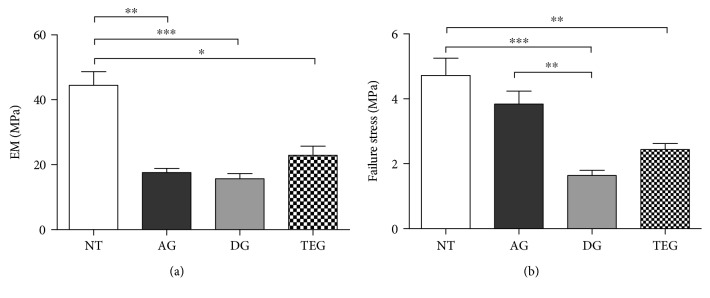
Biomechanical results on the explants at 12 weeks. (a) Elastic modulus of treated samples is significantly lower in NT compared to AG (*p* < 0.01), DG (*p* < 0.001), and TEG (*p* < 0.05); no differences exist between treated tendons. (b) Failure stress is lower between NT and DG or TEG for *p* < 0.001 and *p* < 0.01, respectively; no differences exist between NT and AG; DG has also a lower failure stress compared to AG (*p* < 0.01).

The results of uniaxial tensile tests conducted till failure on NT and tendon substitutes (AG, DG, and TEG) are reported in Figure 9.  Figure 9(a) shows the elastic modulus (EM) at high strains computed in the linear region of each stress-strain curve before the explant failure. The tendons treated with autologous grafts or DG and TEG showed no differences among groups, while a significant decrease of EM existed between the treated tendons and the NT group as expected. Although in the absence of significant differences, an improvement of EM was found in the TEG group compared to both AG and DG. The failure stress is shown in Figure 8(b). No differences were found between AG and NT, while DG and TEG showed a lower failure stress compared to NT for *p* < 0.001 and *p* < 0.01, respectively. Despite no differences were found between TEG and DG, the last one had a significant lower failure stress compared to AG (*p* < 0.01), while no differences were found between AG and TEG.

## 4. Discussion

Tendon ruptures and large defects are great burdens in clinics that always require challenging surgical and grafting procedures. Viable graft materials, as substitutes for tendon repair, have been explored in orthopaedics, and novel biological grafts have been proposed for clinical use. Among biological scaffolds, the use of decellularized tendon-derived matrices represents a promising approach to treat tendon ruptures [7, 8]. In the present study, the efficacy for tendon regeneration of decellularized tendon xenografts enriched or not with autologous bone marrow mesenchymal stem cells via a dynamic culture in bioreactor has been assessed in a rabbit model of full tendon transection and compared to autograft as the gold standard. The rabbit has been chosen as the model of Achilles tendon transection because of its widespread use for tendon engineering purposes due to the appropriate tendon size that allows intravital evaluations (e.g., ultrasounds), the presence of an intrasynovial component and biomechanics similar to flexor tendons of the human hand [8, 20, 26]. The high clinical translatability of this model permitted the evaluation of a regenerative strategy involving the use of decellularized xenografts. Indeed, the ultimate goal was to generate a scaffold free of donor cellular components, while maintaining its biocompatibility. In this context, a cell-based approach for the development of biological vital scaffolds has been extensively demonstrated as an effective strategy [27]. The use of autologous BMSCs was considered because of the well-known capability of these undifferentiated cells in proliferating and differentiating towards tendon-like cells [28], especially when cultured under dynamic stretch and perfusion conditions within the tendon matrix [13]. Indeed, our group has previously demonstrated that the dynamic culture of undifferentiated rbBMSCs injected within the decellularized tendon graft produced a mature and aligned collagen matrix in vitro [13]. This evidence was also supported by others who used different scaffold materials [29–31]. More importantly, the regenerative and immunomodulatory potential of BMSCs has been widely reported in the literature, as well as the capability of BMSCs to secrete growth factors and cytokines for the activation of tissue-resident cells, stimulation of neoangiogenesis, and inhibition of inflammation [32]. Based on these premises, the employed animal model was fundamental to examine the effects on both the integration within the native tissue and the host response of decellularized tendon xenografts enriched with autologous BMSCs specifically cultured in a customized bioreactor. The obtained results demonstrated the beneficial role of undifferentiated BMSCs when loaded within the decellularized xenografts undergoing a stretch-perfusion culture as an immunomodulatory weapon, reducing the inflammatory process commonly associated with nonself, biological implants. An essential role in the evaluation of the host response following the xenograft implantation is played by histological analysis, which permits to investigate not only the morphological changes of the tissue structure but also the inflammatory status and the healing process determined by the treatment. Regarding the recovery and regeneration processes, the modified Stoll's score was able to picture a good assessment of the healing status of implanted grafts 12 weeks after surgery. No significant differences were found between AG and TEG groups that showed a clearly superior tissue healing compared to the DG group. Specifically, the histological results indicate an increased concentration of collagen fibres in both AG and TEG groups, considered as an index of the healing process [33]. Similarly, the presence of a mild inflammatory pattern in these groups represents the basis for the healing process [34]. Indeed, an overlapping between the later stage of inflammation and the proliferation stage in which fibroblasts stat synthesize collagen type III could be possible, then followed by the tardive remodelling phase [35]. These findings were also described by other authors who demonstrated that the inflammatory reaction ameliorates the fibroblastic response, controlling the cell behaviour and the matrix production [3]. In general, AG and TEG showed a similar response in terms of newly formed collagen fibres, mostly type III collagen. Despite type III collagen retains poor biomechanical properties, the early mobilization of the rabbit limbs could convert type III into functional type I collagen over time, as also supported by others [18]. It could also be possible that TEG took the advantage of the effect of both the seeded cells onto the decellularized tendon matrix and the locally injected autologous rbBMSCs, thus resulting in a greater production of collagen compared to unseeded DG [36]. Despite the collagen protein localization and distribution has been assessed in the present study, a quantitative analysis of the collagen gene expression lacks to corroborate this immunohistochemical finding. Morphologically, the cell density increased in all treated tendons as expected, showing both a more physiologically cell distribution and elongated cell morphology in AG compared to TEG. However, although with longer follow-up, the TEG group would probably exert a similar healing process and cell alignment compared to the AG group. Indeed, the migration of resident cells to the repairing site not only comes from the tendon stumps but also comes from the epitenon, thus needing a longer time to reach the injured area [2]. Overall, the higher amount of cells and newly formed collagen fibres could be the reason for larger and thicker tendon structure in the TEG group, as diagnosed by means of ultrasounds and measured on the explants. The increased cellularity in terms of cells able to synthesize tendon-specific ECM is reflected also in the greater level of proteoglycans and glycosaminoglycans detected in AG and TEG groups [37–39], as well as of inflammatory cells in the DG group, thus inducing a common increased swelling. In fact, the DG group demonstrated a massive inflammatory and giant cell response associated with graft destruction and necrosis, as similarly found by others following the implant of extracellular matrix scaffolds [40–42]. This could be due to the use of xenogeneic biological material that in the absence of autologous, immunomodulatory BMSCs determined an adverse immune response of the host. More importantly, the greater level of proteoglycans detected in the DG group compared to the AG and TEG may also be the result of altered mechanical loads that might increase the local levels of growth factor and cytokines, thus affecting also the activation of MMPs and consequently the disorganization of the tendon ECM [38]. Indeed, under physiological conditions, low levels of the latent form of MMPs are present within the matrix and MMPs are activated only during the tissue turnover [24]. Differently, during pathological events, there is an imbalance between the synthesis and degradation of the matrix components (i.e., collagen, glycoprotein, and sGAG) led by the expression and activation of MMPs. In this context, an increase in MMP activity is likely to indicate the matrix degradation, as part of the remodelling process in tissue healing [43]. In particular, MMP-9 commonly participates in collagen degradation, whereas MMP-2 participates in both collagen degradation and remodelling [44, 45]. Despite no differences were found in MMP-2 levels between DG and TEG, it can be hypothesized that in the severely compromised DG group in terms of inflammation and necrosis, MMP-2 could have had a stronger effect in denaturizing both collagen types I and III, thus supporting the immunohistochemical results, i.e., absence of both types of collagen. This phenomenon is particularly true when the analyses are performed in the late time point until the remodelling process occurs [46]. In this context, it would have been helpful to evaluate also the levels of MMP-9, to have a deeper insight into the mechanism driving the collagen degradation process. Notoriously, cells are poorly present in tendons and decellularized tendons are even less populated tissues. Nonetheless, MMPs are difficult to extract being tightly bonded to ECM [47]. For these reasons, the extraction of proteins from the explanted specimens resulted in a demanding process and in a scarce yield, thus representing a limitation of the MMP analysis through gelatine zymography reported in the present study. Indeed, the final concentration of the extracted proteins was certainly sufficient to detect the activity of MMP-2, but not enough to appreciate the levels of MMP-9, as also suggested by Monteiro and colleagues, who loaded only 8 *μ*g of protein to detect MMP-2 and 50 *μ*g to detect MMP-9 with scarce results [48]. Regarding biomechanics, all treated groups had inferior biomechanical properties compared to NT due to the presence of sutures used for the widely employed Kessler's tenorrhaphy [33] that could affect the elasticity of the entire implant, as expected. Moreover, the great presence of collagen type III in AG and TEG, commonly deposited during the tendon healing process, had surely affected the tendon biomechanics [49]. Nevertheless, AG showed a failure stress very similar to the NT. It was also expected that DG and TEG treated tendons depicted a weaker behaviour compared to AG, confirming data reported by others [50, 51]. However, 12 weeks after implantation, we found an increased EM and failure stress in the TEG group compared to the DG, supporting data obtained with all the other analyses. The presence of autologous BMSCs and of a more mature construct obtained from the dynamic culture in OPBS may be responsible for ameliorating the elasticity and resistance of the reimplanted tissue, as also demonstrated elsewhere [52]. Moreover, the absence of a proper amount of both collagen types I and III and of a higher amount of proteoglycans and inflammatory cells in the DG group could have affected the unfavourable biomechanical response in this group, in particular when compared to the cell-reseeded TEG group. Indeed, the presence of a severe local inflammatory response and focal necrosis, together with the great amount of proteoglycans and MMP-2 in the DG group, compromised the collagen deposition with a significant effect not only on the construct structure (histology) but also mainly in the biomechanical properties [53]. Our results are consistent with those of Chong et al. [18] in which intratendinous BMSC therapy improved histological and biomechanical parameters in the early tendon healing of defect models. Otherwise, our findings are in contrast with the study of Zhang et al. [51], in which reseeded decellularized tendon grafts showed similar results of unseeded grafts. The good results obtained in the present study could be explained by utilizing the OPBS to precondition cell-seeded xenografts. Even though this study is limited to the analysis of efficacy at one time point (12 weeks), the use of ultrasound permitted to monitor the implanted grafts also at an early stage of healing (10 days) with a good specificity for lesions and tissue changes, as also supported by others [26]. More importantly, this technique avoided to include a greater sample size and to avoid useless animal sacrifice in order to evaluate the progression of the tendon healing process, thus respecting the 3R's principles. Again, the lack of an internal control for the TEG group—eventually represented by reseeded, engineered grafts without the local injection of BMSCs—did not permit to discriminate the effect of seeded or locally injected cells in terms of both healing and immunomodulatory effect. Finally, further analyses should also monitor the modulation of tissue inhibitors of metalloproteinases (TIMPs) according to MMP levels expressed in the different region of the tendon by means of the reverse zymography method.

Indeed, the evaluation of the balance between MMP and TIMP expression would have been helpful to have a complete picture of the tendon remodelling process driven by the tested regenerative strategies.

## 5. Conclusions

This tissue-engineering-based approach consisting of decellularized tendon xenografts loaded with autologous BMSCs and cultured in a customized stretch-perfusion bioreactor can represent a valid alternative to autografts to repair tendon full defects. Indeed, the results of this study suggest that TEG constructs support the side-to-side tendon repair with the formation of new collagen fibres and biomechanical features similar to the autografts used as controls. Overall, the present study identified an alternative treatment, here represented by the TEG, to the standard use of autograft (AG). Based on the obtained results, TEG could be a valid option in the case of severe tendon ruptures in the presence of tissue loss, showing similar histopathological outcomes both for the TEG and for the AG with respect to the DG group. According to this, TEG could be a suitable graft material as a substitute for tendon reconstruction, bypassing the limited availability of autografts for dimensions and sites of harvest, as well as the consequent donor site morbidity and prolonged surgical time. Conversely, unexpected results occurred in the DG group—in terms of granulation tissue and inflammatory pattern—open new ways to further investigations to ameliorate the decellularization processes and to reduce the abnormal host response to apparently inert decellularized grafts.

## Figures and Tables

**Table 1:** Measurements of width, thickness (mm), and cross-sectional area (mm^2^) of the experimental groups. Data are reported as mean ± standard deviation (SD). A significant difference was found between TEG and DG or NT for width (*p* < 0.05), thickness (*p* < 0.001), and cross-sectional area (*p* < 0.001); a difference was also found between TEG and AG for thickness (^a^*p* < 0.01) and cross-sectional area (^b^*p* < 0.05).

	Width (mm)	Thickness (mm)	Cross-sectional area (mm^2^)
NT	6.74 ± 0.9	5.15 ± 0.6	27.32 ± 5.4
AG	7.41 ± 1.0	5.71 ± 0.6^a^	33.56 ± 7.7^b^
DG	6.41 ± 0.5	4.81 ± 0.4	24.64 ± 7.7
TEG	8.05 ± 0.5^∗^	7.14±0.4^∗∗∗^	45.20±5.2^∗∗∗^

**(a):** 

	ECM organization (0-2)	Cellularity (0-2)	Cell alignment (0-2)	Cell distrib. (0-1)	Cell nuclei morphol. (0-2)	Proteoglycans (0-1)
AG	1.07	0.88	1.00^a^	0.58	0.77	0.25
DG	0.56^∗^	0.32^∗^	0.25^a^	0.14^∗^	0.33	0.22^b^
TEG	1.06	0.86	0.72	0.56	0.89	0.50^b^

**(b):** 

	Degenerative changes (0-3)	Vascularization (0-1)	Inflammation (0-1)	Org. tissue repair (0-2)	Transition zone (0-2)
AG	2.03	0.58	0.62	1.02	1.30
DG	1.10	0.06^∗^	0.08^∗^	0.46	0.33^∗^
TEG	1.75	0.51	0.49	0.97	1.17

## Data Availability

The data used to support the findings of this study are available from the corresponding author upon request.
